# Эффективность стандартных методов в лечении пролактин-секретирующей карциномы гипофиза

**DOI:** 10.14341/probl13401

**Published:** 2024-11-05

**Authors:** Л. И. Астафьева, П. Л. Калинин, Ю. Ю. Трунин, Г. Л. Кобяков, Ю. Г. Сиднева

**Affiliations:** Национальный медицинский исследовательский центр нейрохирургии им. акад. Н.Н. Бурденко; Национальный медицинский исследовательский центр нейрохирургии им. акад. Н.Н. Бурденко; Национальный медицинский исследовательский центр нейрохирургии им. акад. Н.Н. Бурденко; Национальный медицинский исследовательский центр нейрохирургии им. акад. Н.Н. Бурденко; Национальный медицинский исследовательский центр нейрохирургии им. акад. Н.Н. Бурденко

**Keywords:** агрессивные опухоли гипофиза, карцинома гипофиза, метастазы, гипофизарная нейроэндокринная опухоль, каберголин, темозоломид

## Abstract

Карцинома гипофиза (метастатическая нейроэндокринная опухоль гипофиза) — опухоль гипофиза с подтвержденными краниоспинальными и/или системными метастазами. Эти опухоли встречаются редко, составляя всего 0,1–0,5% новообразований гипофиза, и характеризуются высокой смертностью. В представленном случае у молодого пациента с 25-летним анамнезом агрессивной многократно рецидивирующей пролактин-секретирующей опухоли гипофиза с последующей ее трансформацией в карциному гипофиза с интракраниальным метастазированием на фоне стандартной терапии (удаление метастаза, лучевое лечение и терапия каберголином) отмечена длительная ремиссия заболевания.

## Abstract (en)

Pituitary carcinoma (metastatic neuroendocrine tumor of the pituitary gland) is the pituitary tumor with confirmed craniospinal and/or systemic metastases. These tumors are extremely rare accounting for only 0.1% to 0.5% of all pituitary tumours and are characterized by high mortality. In the presented case, pituitary carcinoma with intracranial metastasis was diagnosed in a young patient 25 years after manifestation of an aggressive recurrent prolactin-secreting pituitary tumor. Standard therapy (removal of metastasis, radiation therapy, cabergoline therapy) resulted in a long-term remission of the disease.

## АКТУАЛЬНОСТЬ

Карцинома гипофиза (КГ, метастатическая нейроэндокринная опухоль гипофиза — PitNET) — опухоль гипофиза с подтвержденными краниоспинальными и/или системными метастазами [1][2]. КГ встречаются редко, составляя всего 0,1–0,5% новообразований гипофиза. Заболеваемость КГ составляет 1–4 случая на 1 млн населения. Однако эти цифры могут быть занижены, поскольку до 75% исторических диагнозов КГ были поставлены только при посмертном вскрытии. КГ обычно манифестируют на 4–6-м десятилетии жизни со средним возрастом на момент постановки диагноза 45 лет. Описаны редкие случаи КГ и в детском, и пожилом возрасте [3][4].

Анализ ранее опубликованных случаев КГ указывает на высокую смертность (66%) в течение 1 года после постановки диагноза КГ в исследовании Pernicone PJ [5], 55% случаев в исследовании Yoo F, при этом среднее время от диагностирования КГ до смерти составляло всего 10 месяцев [6].

В представленном клиническом наблюдении у молодого мужчины лактотрофная КГ с интракраниальным метастазированием диагностирована через 15 лет после хирургического и лучевого методов лечения агрессивной рецидивирующей опухоли гипофиза. Последующее хирургическое и лучевое лечение метастазов и назначение адекватной дозы каберголина привело к длительной ремиссии заболевания.

## ОПИСАНИЕ СЛУЧАЯ

Мужчина 35 лет в ноябре 2015 г. обратился с жалобами на головные боли, опущение правого верхнего века.

Из анамнеза: в 1998–1999 гг. (впервые в возрасте 18 лет) был трижды оперирован транскраниальным доступом в различных клиниках по поводу рецидивирующей опухоли правого кавернозного синуса, вызывающей глазодвигательные нарушения (двоение и птоз справа), расцененной по клинико-рентгенологическим данным первоначально как менингиома кавернозного синуса. Результат гистологического диагноза (от 3-й операции, проведенной в НМИЦ нейрохирургии им. ак. Н.Н. Бурденко): аденома гипофиза с митозами (иммуногистохимическое исследование не проводилось).

После третьей операции впервые было проведено гормональное исследование крови и выявлено повышение уровня пролактина (ПРЛ) более 10 000 мЕд/л (30–360). Получал терапию бромокриптином в течение 1,5 года (доза препарата не известна), однако в связи с отсутствием снижения уровня ПРЛ прием препарата самостоятельно прекратил. В январе 2000 г. проведен курс дистанционной гамма-терапии на область турецкого седла (РОД 1,8Гр, СОД 49,8Гр), далее к врачам не обращался.

В 2015 г. в связи с появлением жалоб на головные боли было проведено МРТ-исследование головного мозга, выявившее объемные образования правой височной области и области задней поверхности пирамиды височной кости, убедительных данных за продолженный рост опухоли в области правого кавернозного синуса нет (рис. 1).

Госпитализирован в НМИЦН для проведения нейрохирургического лечения. В неврологическом статусе: общемозговая симптоматика, очаговая симптоматика в виде грубого пареза III нерва справа, недостаточности V нерва справа, а также зрительные нарушения (острота зрения OD-0,2, OS-1,0, битемпоральная гемианопсия) в виде поражения правого зрительного нерва.

Гормональный анализ крови: ПРЛ — 62 521 мЕ/л (45–375), свТ4 — 6,2 пмоль/л (11,5–22,7), ТТГ — 0,53 мЕд/л (0,4–4,0), кортизол — 30 нмоль/л (119–618), тестостерон — 0,0 нмоль/л (12–35), ЛГ 0,7 Е/л (15–9,3), ФСГ 1,1 Е/л (1,4–18,1), инсулино-подобный фактор роста 1 — 55,9 нг/мл (115–307). Диагностированы гиперпролактинемия, пангипопитуитаризм (гипотиреоз, гипокортицизм, гипогонадизм, СТГ-дефицит). В дооперационном периоде назначена терапия препаратами гидрокортизона, левотироксина.

02.11.15 г. проведено удаление опухоли правой височной доли. Гистологический диагноз: фрагменты обильно васкуляризированной солидной опухоли, состоящей из крупных клеток с округлыми ядрами, имеющих тенденцию к формированию периваскулярных структур, отмечаются митозы. Иммуногистохимическое исследование выявило экспрессию ПРЛ, синаптофизина, хромогранина А, виментина (в сосудах, строме и капсуле), CD34 (в сосудах), ИМ Ki 67 до 10%. С учетом локализации опухолевого очага, морфологической картины и иммунофенотипа опухоли диагностирован метастаз пролактин-секретирующей карциномы гипофиза.

Пациенту была проведена ПЭТ-КТ с 18F-ФДГ — получены данные о наличии активной опухолевой ткани в костях основания черепа справа. Других очагов патологического накопления радиофармпрепарата не выявлено (рис. 2).

После операции отмечено снижение уровня ПРЛ до 53 538 мЕд/л, начата терапия каберголином в дозе 1 мг с постепенным повышением до 2 мг в неделю. Отмечена нормализация ПРЛ (380 мЕд/л) в августе 2016 г. По данным МРТ не отмечено продолженного роста опухоли (рис. 3).

В январе 2016 г. проведена адъювантная стереотаксическая радиотерапия на роботизированном линейном ускорителе CyberKnife: к очагам в области проведенной операции и в базальных отделах правого и левого полушарий головного мозга подведено за 6 фракций СОД 85 Гр (суммарно на все очаги).

С декабря 2017 г. к таблетированной терапии гидрокортизоном и левотироксином добавлен трансдермальный гель тестостерона. При ежегодных контрольных осмотрах с 2018-го по 2023 гг. рецидива опухоли не выявлено (рис. 4). На фоне приема каберголина 2 мг в неделю сохраняется нормопролактинемия (ПРЛ — 149 мЕд/л). Состояние пациента удовлетворительное, он самостоятельно себя обслуживает и ведет обычный образ жизни.

**Figure:**
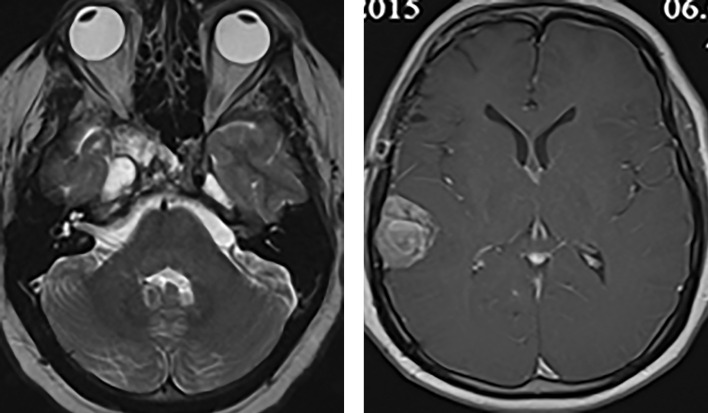
Рисунок 1. МРТ головного мозга пациента С. 35 лет визуализируются опухоли правой височной области и области задней поверхности пирамиды височной кости.

**Figure:**
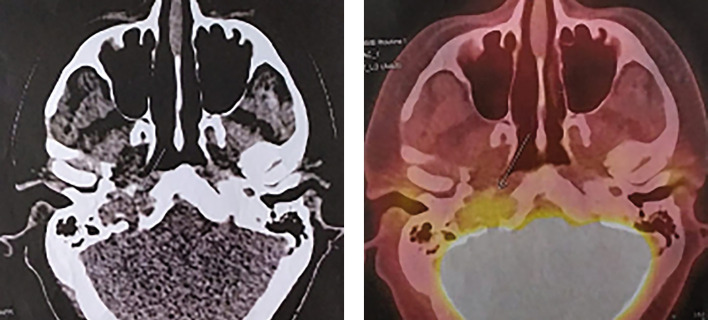
Рисунок 2. На серии ПЭТ/КТ выявляются очаги патологического накопления 18F-ФДГ: в большом крыле клиновидной кости справа с макс SUV 4,85, по КТ — мягкотканное образование, деструирующее клиновидную кость и прилегающие участки ската и пирамиды правой височной кости с образованием внекостного компонента, распространяющегося интракраниально и дистально по ходу внутренней сонной артерии до уровня С1, размерами около 2,8х1,5 см (указано стрелкой).

**Figure:**
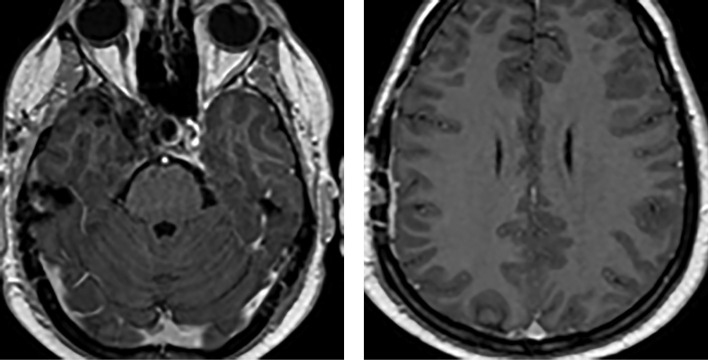
Риcунок 3. МРТ (январь 2016 г.) — данных за рецидив опухоли не выявлено.

**Figure:**
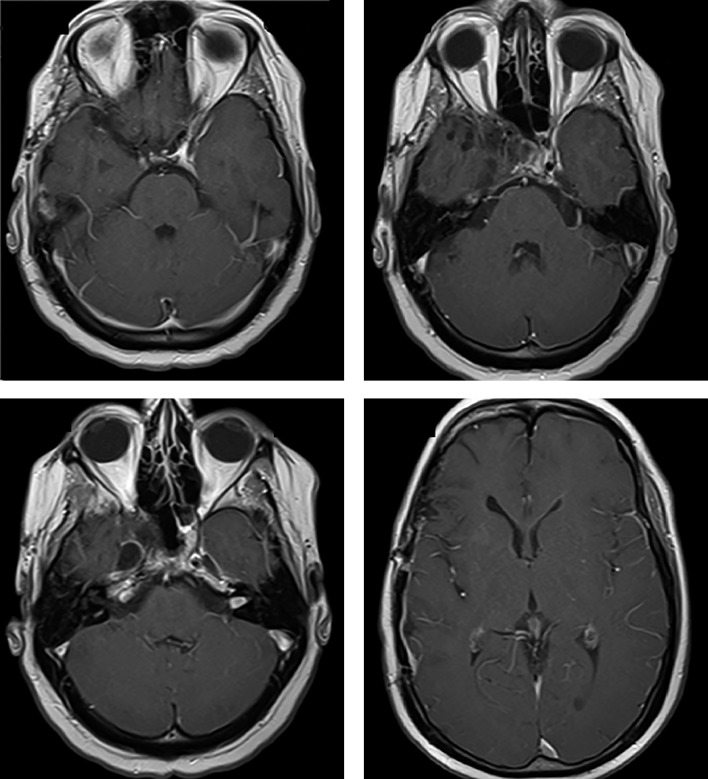
Рисунок 4. МРТ (2020–2023 гг.) — не отмечено продолженного роста опухоли.

## ОБСУЖДЕНИЕ

Интервал между диагностикой аденомы гипофиза и началом ее агрессивного поведения в литературе очень вариабелен: некоторые опухоли проявляют агрессивные черты с момента постановки диагноза, в то время как в других случаях опухоль может находиться в «состоянии покоя» в течение многих лет с последующим быстрым ростом и даже развитием метастазов [2]. Представляют ли такие изменения трансформацию de novo в злокачественную опухоль или результат медленного прогрессирования опухоли, предрасположенной к агрессивному поведению, является предметом дискуссий [3].

В недавно опубликованном исследовании Европейского общества эндокринологов (ESE, 2022 г.) было включено 50 пациентов с КГ. Средний возраст при постановке диагноза КГ составлял 48 лет, и эти опухоли преобладали у мужчин. КГ представляли в большинстве случаев гормонально-активные опухоли гипофиза; преобладающими были кортикотрофные и лактотрофные типы опухолей, которые выявлялись в 38 и 32% случаев соответственно [7].

Начальная клиническая картина КГ обычно отражает инвазивный характер опухоли; большинство клинических проявлений являются результатом «масс-эффекта» опухоли, включая головные боли, нарушения зрения, глазодвигательные нарушения. Диагнозу КГ могут предшествовать появление таких редких симптомов, как потеря слуха, атаксия, двигательные нарушения или опухоли в области шеи, что требует дальнейшего диагностического поиска. Метастазы редко доминируют в клинической картине на ранних стадиях заболевания, а иногда обнаруживаются только после смерти [3].

Среднее время обнаружения метастазов после постановки диагноза опухоли гипофиза составляет от 5 до 9 лет (диапазон 0,5–34 года). Насколько известно, нет сообщений о метастатических поражениях, предшествующих диагностике опухоли гипофиза [3].

Метастатическое распространение обычно включает ЦНС (головной, спинной мозг, твердую и мягкую мозговую оболочку) и отдаленные органы, такие как скелет и печень, реже — легкие и шейные лимфатические узлы. ЦНС является первой локализацией метастазов примерно у половины пациентов. В анализе, проведенном Yoo F, в 43% случаев метастазы были интракраниальными, в 37,5% — спинальными. Метастазы в печень наблюдались в 14%, в шейные лимфатические узлы и кости — в 11 и 10% случаев соответственно [6].

В настоящее время руководство ESE рекомендует выполнять расширенную оценку для поиска вторичных локализаций с использованием визуализации (КТ или МРТ) и/или функциональной визуализации (ПЭТ/КТ с 18F-фтордезоксиглюкозой и с Ga68-DOTA-ТАТЕ в случаях со специфическими симптомами (неврологические жалобы или боли в шее и/или спине) или с явными несоответствиями между биохимическими и рентгенологическими данными [2].

Ранняя идентификация опухолей гипофиза с риском прогрессирования в КГ в настоящее время невозможна, и никакая комбинация гистологических, иммуногистохимических или ультраструктурных признаков не могут распознать аденому гипофиза, которая будет прогрессировать в карциному [3][7].

Основной целью гистологической оценки КГ является подтверждение гипофизарного происхождения метастазов. Биопсия метастазов КГ особенно важна для дифференциального диагноза с метастатическим поражением негипофизарного злокачественного новообразования, тем самым влияя на прогноз и лечение.

Первоначально в лечении КГ применяют стандартные методы: хирургические, лучевые и медикаментозные. При лактотрофных КГ в качестве первичного метода используется каберголин в максимально переносимых дозах. Однако в большинстве случаев КГ резистентны к этой терапии, и требуется назначение химиотерапии, препаратом выбора которой является темозоломид [2].

В настоящее время показано, что пациенты, которые реагируют на темозоломид, имеют явное преимущество в выживаемости. Однако даже при лечении темозоломидом в исследовании ESE (2022 г.) медиана выживаемости после обнаружения метастазов составила всего 5,1 года (95% ДИ, 2,7–7,5) [8].

В представленном нами наблюдении у молодого мужчины заболевание манифестировало инвазивной опухолью гипофиза с преимущественным врастанием в кавернозный синус, и расцененной вследствие этого как менингиома кавернозного синуса. В клинической картине доминировали глазодвигательные нарушения вследствие одностороннего опухолевого поражения III и V нервов. Учитывая быстрый рост опухоли, пациент был трижды оперирован транскраниальным доступом в течение 1,5 года с момента диагностирования заболевания. Несмотря на отсутствие как своевременной гормональной диагностики, так и адекватной терапии агонистами дофамина, последующее после операции лучевое лечение привело к длительной стабилизации заболевания. Рецидив был диагностирован через 15 лет в виде манифестации интракраниальных метастазов с их гистологически подтвержденным гипофизарным генезом с экспрессией ПРЛ и высокими митотическим и пролиферативным индексами.

Последующая после операции терапия каберголином привела к стойкой нормализации уровня ПРЛ. Учитывая злокачественный характер опухоли, через 6 мес после операции проведена стереотаксическая радиотерапия. На фоне продолжения терапии каберголином сохраняется нормопролактинемия с отсутствием рецидива опухоли в течение всего последующего 8-летнего периода наблюдения. Тем не менее, учитывая возможный рецидив опухоли, пациент нуждается в постоянном динамическом наблюдении.

## Заключение

Таким образом, несмотря на появление современных (темозоломид) и экспериментальных методов (пептидной рецепторной радионуклидной терапии, молекулярно-таргетной и иммунотерапии) [9], стандартные методы лечения аденом гипофиза, в частности каберголин, могут быть эффективны и в лечении КГ.

Учитывая редкую встречаемость этой патологии и малочисленность клинических исследований, наблюдение и выбор оптимального метода лечения пациентов с агрессивными опухолями и карциномами гипофиза на каждом этапе заболевания должен осуществляться междисциплинарной командой врачей-экспертов, обладающих необходимой квалификацией и имеющих опыт лечения этой патологии.

## Дополнительная информация

Источники финансирования. Работа выполнена по инициативе авторов без привлечения финансирования.

Конфликт интересов. Авторы декларируют отсутствие явных и потенциальных конфликтов интересов, связанных с содержанием настоящей статьи.

Участие авторов. Все авторы одобрили финальную версию статьи перед публикацией, выразили согласие нести ответственность за все аспекты работы, подразумевающую надлежащее изучение и решение вопросов, связанных с точностью или добросовестностью любой части работы».

Согласие пациента. Пациент добровольно подписал информированное согласие на публикацию персональной медицинской информации в обезличенной форме (в журнале «Проблемы эндокринологии»).
